# Microbial proteomics: a mass spectrometry primer for biologists

**DOI:** 10.1186/1475-2859-6-26

**Published:** 2007-08-15

**Authors:** Robert LJ Graham, Ciaren Graham, Geoff McMullan

**Affiliations:** 1 School of Biomedical Sciences, University of Ulster, Coleraine, County Londonderry, BT52 1SA, UK

## Abstract

It is now more than 10 years since the publication of the first microbial genome sequence and science is now moving towards a post genomic era with transcriptomics and proteomics offering insights into cellular processes and function. The ability to assess the entire protein network of a cell at a given spatial or temporal point will have a profound effect upon microbial science as the function of proteins is inextricably linked to phenotype. Whilst such a situation is still beyond current technologies rapid advances in mass spectrometry, bioinformatics and protein separation technologies have produced a step change in our current proteomic capabilities. Subsequently a small, but steadily growing, number of groups are taking advantage of this cutting edge technology to discover more about the physiology and metabolism of microorganisms. From this research it will be possible to move towards a systems biology understanding of a microorganism. Where upon researchers can build a comprehensive cellular map for each microorganism that links an accurately annotated genome sequence to gene expression data, at a transcriptomic and proteomic level.

In order for microbiologists to embrace the potential that proteomics offers, an understanding of a variety of analytical tools is required. The aim of this review is to provide a basic overview of mass spectrometry (MS) and its application to protein identification. In addition we will describe how the protein complexity of microbial samples can be reduced by gel-based and gel-free methodologies prior to analysis by MS. Finally in order to illustrate the power of microbial proteomics a case study of its current application within the Bacilliaceae is given together with a description of the emerging discipline of metaproteomics.

## Background

**Figure 1:**
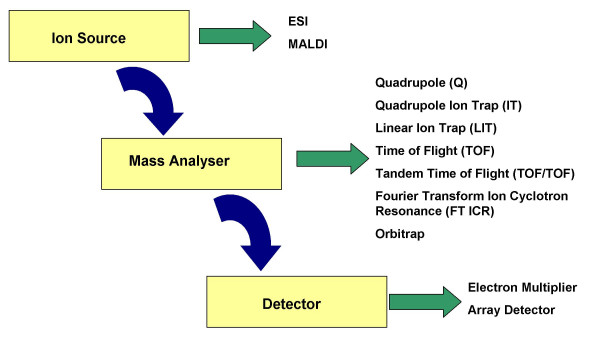
The three principle components of the mass spectrometer with examples of each.

Mass spectrometry has its origins in the studies performed by J. J. Thomson and his student F. W. Aston around the turn of the last century [1]. An advantage of mass spectrometers over other analytical instruments is that it affords a high degree of accuracy (~0.01–0.001%) and sensitivity (detection of 10^-9 ^– 10^-18 ^mol of sample required) when determining the molecular weight of biological compounds [2]. A mass spectrometer is an instrument that produces ions from a sample, separates them according to their mass-to-charge ratio (m/z) and records the relative abundance of each of the ions to obtain a mass spectrum [3]. The mass spectrometer may be broken down into three principal components; the ion source, mass analyser and the detector (Figure 1). Until relatively recently mass spectrometry was restricted in its use to determining the molecular weight of relatively volatile compounds. The development of 'soft ionisation' techniques in the 1980s by Fenn *et al *permitted the ionisation and vaporization of large, polar, and thermally labile biomolecules such as proteins and peptides that previously did not lend themselves to such analytical techniques [2,4]. Soft ionisation refers to the ability to ionise and volatilise thermally labile compounds, such as peptides, without inducing fragmentation [2]. The characterisation and quantification of proteins has been greatly enhanced by the development of two critical 'soft ionisation' technologies namely electrospray ionization mass spectrometry (ESI-MS) and matrix-assisted laser desorption ionization time of flight mass spectrometry (MALDI-TOF MS). Both these techniques have had immense importance in the field of biological and pharmaceutical science so much so that one quarter of the 2002 Nobel Prize for Chemistry was awarded to both John Fenn and Koichi Tanaka for their revolutionary work in ESI and MALDI respectively.

### Electrospray Ionisation (ESI)

**Figure 2:**
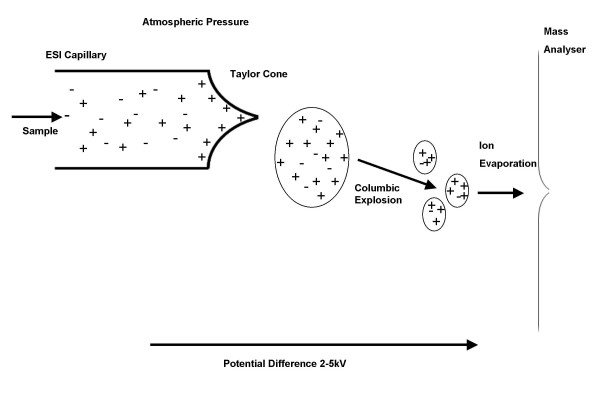
Simplified representation of the process of ESI.

The pioneering work of Fenn *et al *in the 1980s [4] heralded the development of electrospray ionisation for mass spectrometry (ESI-MS) of large biomolecules. ESI has become a mainstream method for life science research as a result of its high sensitivity and broad applicability. ESI is typically carried out in tandem with high performance liquid chromatography (HPLC) usually for proteomic applications in conjunction with a nano electrospray conformation [5]. ESI generates charged microdroplets containing analyte ions. The sample of interest is dissolved in a solvent and then pumped through a thin capillary or needle that is raised to a high potential that may be positive or negative. As a result of the electric field the solution exits the tip of the capillary in the shape of a cone, known as the Taylor cone [6]. At the apex of this cone charged droplets are sprayed from the capillary when the electrostatic repulsion of the charged molecules approaches the surface tension of the solution. These small charged droplets travel down a pressure and potential gradient towards an orifice in the mass-spectrometer. As the droplets traverse this path they become desolvated and reduced in size however their charge remains constant [7]. As the droplet shrinks this increases the electrostatic stress near the surface of the droplet. The droplet can no longer sustain the charge when the force of electrostatic repulsion between like charges becomes equal to the surface tension force known as the Rayleigh stability limit. At this juncture the droplet undergoes Coulombic fission leading to the production of smaller droplets. This process continues until the point is reached that either an ion desorbs from the droplet or the solvent is completely removed [1] (Figure 2). The gas phase ions are then detected as a series of multiply charged ions. To determine the molecular weight (Mr) of the compound, a simple algorithm transforms this ion series into a single Mr value. Under ESI, macromolecules such as proteins and peptides yield multiply charged ions (e.g. [M+nH]^n+^). Electrospray ionisation is typically characterised as a concentration sensitive methodology where signal strength is proportional to concentration. This holds true at μl min^-1 ^flow rates however, at very low flow rates of <100 nl min^-1 ^extremely efficient ionisation occurs and signal strength is proportional to the absolute quantity of analyte present [7].

### Matrix-Assisted Laser Desorption-Ionisation (MALDI)

**Figure 3:**
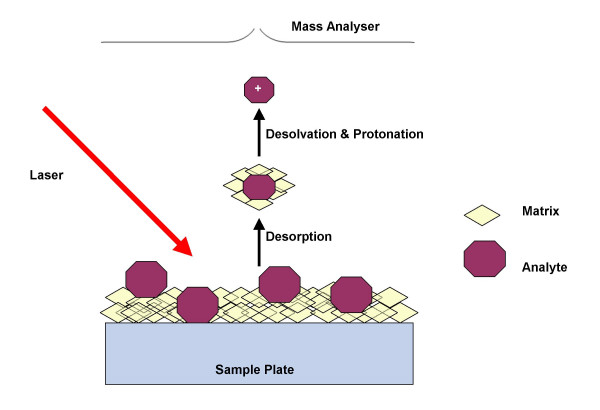
Simplified representation of the process of MALDI.

Matrix-assisted laser desorption-ionisation (MALDI) is a method that was pioneered by Karas and Hillenkamp when in 1987 they utilised an ultraviolet laser to desorb intact molecular ions of proteins that were co-crystallised in a nicotinic acid matrix solution [8,9]. Unlike ESI generally only singly charged ions are observed for MALDI. In MALDI the analyte of interest is co-crystallised with an excess of matrix, that is utilised as a diluent preventing the analyte from forming large aggregates that would otherwise be too large to desorb [10,11]. The matrix also absorbs UV light from a laser thus facilitating analyte desorption and ionisation [10]. There are a number of different matrixes that may be used in MALDI-MS. Typically for protein analysis 3,5-dimethoxy-4-hydroxycinnamic acid (sinapinic acid) is the standard matrix used whilst α-cyano-4-hydroxycinnamic acid (CHCA) is often used when analysing peptides. Analyte and matrix are both spotted onto a metal target plate which is then inserted into a high vacuum source region within the mass spectrometer [11]. The target plate is subjected to laser bombardment and analyte molecules are vaporised along with the matrix molecules. The process of desorption and ionisation in MALDI is not fully understood with several influencing factors such as, laser wavelength, pulse width and chemical properties of the matrix and analyte [12]. During laser irradiation a gas jet of matrix neutrals and surrounding analyte molecules is formed (Figure 3). The matrix molecules are strongly excited at this stage and analyte molecules are thought to be ionised as a result of multi-step interactions with the matrix resulting in proton transfer giving both protonated and deprotonated analyte ions [10,11].

### Mass Analysers

There are several different types of mass analysers available commercially (Figure 1). Each mass analyser will have intrinsic advantages and disadvantages over the other types and all differ in their mode of operation and ability to carry out particular types of analyses. Instrument performance of all the mass analysers is dependent on the analyte and the experimental setup of the analyses to be undertaken. The choice of mass analyser will ultimately be decided by the throughput needs of the research to be undertaken, the cost of the machine and funds available to the individual research group. Below is a brief overview of the principles and standard mass analysers used in proteomics, for further background information readers are invited to read the following comprehensive reviews [13,14].

### Quadrupole Mass spectrometers

The single quadrupole mass analyser consists of four circular metal rods placed equidistance from each other with an oscillating electric field applied through a combination of direct current (DC) and radio frequency (RF) to the rods. One pair of rods are supplied with a positive DC and RF voltage, whilst the second pair are supplied with a negative DC and RF voltage 180° out of phase with the first pair. This creates a quadrupolar electric field and for a particular amplitude of direct current and radio frequency only ions of a given mass to charge (*m*/*z*) ratio will have a stable trajectory and therefore be able to pass through to the detector, all other ions will collide with the quadrupole rods. By adjusting the DC and RF voltages, ions of different *m*/*z *values can pass through to the detector. The ramping of these parameters can occur in less than 1/6th of a second allowing ions over a wide *m*/*z *range to be scanned in the one mass spectrometry experiment. Today however it is unusual to see large scale proteomic or metabolomic studies carried out using a single quadrupole instrument. More typically the instruments of choice will be the hybrid quadrupole ion trap mass spectrometer or a triple quadrupole mass spectrometer. Triple quadrupole mass spectrometers allow for tandem mass spectrometry and consist of three sections (Q1, Q2 and Q3). Q1 acts as a mass filter allowing only ions of a certain mass to move further into the mass spectrometer. Q2 functions as a collision cell for fragmentation of the ions. Q3 acts as a second mass separating quadrupole allowing the fragment ions to be separated and resolved before they are measured by the detector [5].

### Ion Trap Mass spectrometers

In the ion trap analyser, a rotating three-dimensional electrostatic field effectively captures the ion of interest. The trajectories of simultaneously trapped ions of consecutive specific mass/charge ratio become sequentially unstable and leave the trapping field in order of their mass/charge ratio. Ion trap analysers have the advantage of fast data acquisition with excellent sensitivity [13]. One of their main characteristics is the ability of the ion trap to carry out multiple fragmentation of precursor and product ions, a process called tandem mass spectrometry (MS/MS). In such experiments, all ions except for that under investigation are ejected form the field, the remaining ion is fragmented and the product ions sequentially released to be further analysed at the detector [8]. Ions can be trapped, fragmented and analysed several times in a multi-stage process known as MS^n^. The actual amount of MS^n ^steps that can be achieved is dependent on the instrument used although MS^3 ^and MS^4 ^are typical with up to MS^12 ^being reported in the literature [15]. The drawbacks of using this analyser are it has limited resolution, poor trapping efficiencies and low mass accuracy due to space charging effects [13]. The development of linear ion trap analysers by the scientific community was an attempt to overcome these inherent problems. Linear ion traps have enhanced ion trapping capacities, and a larger volume than 3D traps allowing more ions to be stored before space charge effects are seen [16].

### Time of Flight

A time of flight (TOF) mass spectrometer is one of the simplest analysers available wereby ions are accelerated by an electric field into a long, straight, evacuated tube prior to detection. The distance of the tube to the detector is fixed and the ions are accelerated to the same energy. As the ions will have different velocities after they are accelerated they will be separated in space and time [17]. As the ions have the same kinetic energy, the smaller the mass the faster the ion will transverse the tube. The time taken for the ion to traverse the tube is therefore proportional to its mass to charge ratio with each ion having a characteristic time of flight [5,18].

### Tandem Mass Spectrometry (MS/MS)

**Figure 4:**
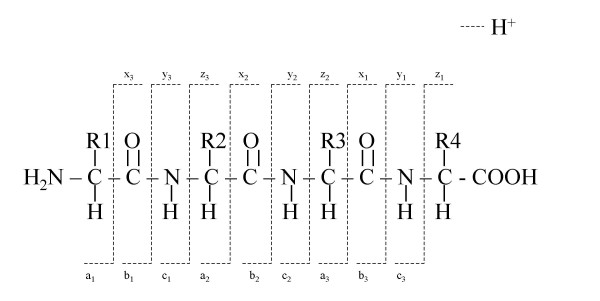
Ions Generated from Fragmentation of Peptide/Proteins.

In order to acquire structural or peptide sequence information, it is necessary to induce fragmentation of the peptides of interest. This is not possible with soft ionisation techniques such as ESI and MALDI, however, the use of these techniques in conjunction with tandem mass spectrometry (MS/MS) has allowed the structural elucidation of a wide range of peptides. MS/MS spectra of peptides however, are often complex and difficult to interpret [19]. In MS/MS peptides are individually ionised in the source region using ESI or MALDI. These peptides are then further separated, based on their *m*/*z *ratio. The selected ions are allowed into a collision cell, which is filled with an inert gas such as xenon, argon or nitrogen, collisions then occur between the precursor ion and inert-gas atoms (molecules). In these collisions part of the precursor ion's translational energy can be converted into internal energy, and as a result of single or multiple collisions an unstable excited state is populated. Excited precursor ions decompose to produce product ions in a process termed 'collision-induced dissociation' (CID) [1]. The types of fragment ions observed in a MS/MS spectrum depend on many factors that include the primary sequence of the peptide, the amount of internal energy and the charge state [20]. The main types of ions observed in the fragmentation of protonated peptides are well established noting that fragments can only be detected if they carry a charge. If the charge is retained on the N-terminal fragment the ion is classified as a, b, or c and x, y, or z if the charge is carried on the C-terminal. The nomenclature for fragmentation ions is described by Johnson *et al *[20] and is shown in Figure 4. These product ions are then mass analysed producing a spectra [1]. The tandem mass spectrometry data can be used to elucidate the primary sequence of a peptide. The process of deducing an amino acid sequence from the tandem mass spectra is aided by the ready availability of protein and DNA databases [21].

### Other Mass Spectrometers

Other commercially available mass spectrometers include the Fourier transform ion cyclotron (FT-ICR) mass spectrometer, an extremely high resolution instrument that determines ion masses very accurately with low attomole sensitivity for proteins [22]. However, the expense of such an instrument has limited its availability within the academic world. Another mass spectrometer that is extensively used in proteomics is the Q-TOF, a hybrid combining a triple quadropole instrument with a reflector TOF in place of Q3. This instrument is considerably less expensive than the FT-ICR whilst still having a wide dynamic range and also a high degree of mass accuracy, >5 ppm in MS/MS mode [14]. The orbitrap mass analyser is the first new mass analyser to be introduced commercially for three decades [13]. The orbitrap traps ions not using magnets or radio frequency but using electrostatic fields [23]. The orbitrap like the FT- ICR mass spectrometer affords high mass resolution and high mass accuracy experimentation [24]. This instrument is only recently commercially available however its relatively compact size and lower cost may make this analyser attractive to academia.

### Proteomics

Proteomics may be defined as the analysis of the entire protein complement expressed in a cell or any biological sample at a given time under specific conditions [25]. The field can be split into two areas, expression proteomics and functional proteomics, the former aims to measure differential expression of proteins within a cell under varying conditions while the latter seeks to characterise the components of cellular compartments, multiprotein complexes and signalling pathways [5]. Unlike DNA microarray analysis, proteomics currently does not have the equivalent of the polymerase chain reaction to enhance the signal, making proteins of low copy number difficult to detect. Developments in the ability to study gene expression at the genome level have been complemented by the development of high throughput multi-dimensional methods for proteome analysis [26].

Mass spectrometry has greatly enhanced research in the field of microbial proteomics. In the areas of global microorganism identification through intact cell mass spectrometry; identification of membrane, cellular, periplasmic and extracellular proteins; full proteome expression in organisms (2D-PAGE coupled to MS and 2D LC coupled to MS); differential protein expression levels under stress and non stress conditions and identification of posttranslational modifications of proteins within organisms [27].

#### Top Down Proteomics

The top down strategy was first introduced by McLafferty and colleagues utilising the immense analytical power of FT-ICR MS [28]. The goal of this methodology is to identify intact proteins utilising mass spectrometry, without the need for prior proteolytic digestion of the sample. Significantly, the protein also need not be purified to homogeneity. Initially proteins are introduced into the mass spectrometer in the gas phase and are then fragmented. The fragmentation profile generated is then analysed and compared with a specifically designed database in order to identify the proteins present [29-31]. The methodology is not as widely used as peptide fragmentation and usually requires a high resolution mass spectrometer such as FT-ICR [28], Maldi/TOF-TOF [31] or Q-TOF [29,32]. This methodology has, however, been used successfully for microbial proteomics in the analysis of *Bacillus *spores in order to ascertain the species that the spore was derived from [31]. In addition it has also been used for the identification of pathogenicity biomarkers from a comparison of 12 strains of *Enterobacter sakazakii *[32]. It should be noted that the classification of top-down proteomics has recently been widened to include the multidimensional separation (gel based or LC based) of undigested protein samples followed by tryptic digestion of isolated proteins and subsequent analysis of peptides by MS [33].

#### Bottom Up Proteomics

This approach refers to any methodology that identifies proteins from the analysis of peptides derived from the proteolytic digestions of those proteins [34]. The resultant peptide mixture is fractionated by chromatography before being subjected to tandem mass spectrometry. The fragmentation pattern from each peptide produces a peptide sequence tag and the resultant data is analysed by bioinformatics tools and searched through amino acid or protein databases in order to identify the protein [30]. The simplest form of this approach is known as 'shotgun proteomics'. This refers to the direct analysis of a complex protein mixture without fractionation. The complex mixture is enzymatically digested to produce peptides, this peptide mixture is then fractionated on a reverse phase C_18 _column before analysis on the mass spectrometer. This methodology gives a rapid large scale global analysis of the protein mixture, however, it gives limited penetration into the proteome [35,36]. The effectiveness and proteome coverage of shotgun analysis has been greatly enhanced by coupling it with multidimensional separation techniques [36].

### Proteomic techniques for the large scale analysis of microorganisms

Until recently, the study of global protein expression was performed nearly exclusively using two-dimensional gel electrophoresis (2D PAGE), a technique developed in the 1970s [37] with significant advances in the intervening decades. For a detailed description of the current status of this technology the reader is directed to the excellent review by Gorg et al [38]. The strength of 2D PAGE is that it can separate up to 10,000 proteins in one gel [39]. Every component is fractionated on the first dimension, by isoelectric focusing and then further resolved according to molecular weight in the second dimension [34,37]. At this point in the proteomic workflow a snapshot of the organism/cell may be visualized. An emerging trend is to deposit images of these 2D gels with databases such as Swiss-2D PAGE or Gelbank as reference material [40]. The usefulness of such repositories is yet to be demonstrated. A limitation of 2D PAGE is typically many more spots are resolved on the gel than are actually identified by the researchers involved [41,42]. This is as a result of a second analytical step that must be employed in order to identify the proteins present. Proteins are excised from the gel, subjected to proteolytic digestion, and identified or sequenced; this step is usually carried out manually and is very time-consuming [39,41] although the advent of computerised gel visualisation and robotic spot excision equipment has gone some way to alleviate these 'bottle necks' [38]. The 2-D PAGE methodology has traditionally had a number of practical limitations that the researcher should be aware of with the main issue being the wide dynamic range of proteins present within a biological sample thus proteins present in low copy numbers, and therefore low concentration, are often not visualised on 2-D PAGE gels [39,41]. A number of additional limitations can be encountered such as: most isoelectric focusing gels can only focus proteins between the pI ranges 3–10, so proteins with extreme pI will not be seen on the gels; however protocols have been developed to allow separation and then visualisation of highly alkaline proteins with a pI up to 12 [38]; Most 2-D PAGE gels cannot resolve proteins smaller than 10 kDa and above 200 kDa. Due to the nature of the buffers used in isoelectric focusing the range of solubilising detergents that can be used in this methodology are restricted, thus making it difficult to solubilise certain membrane proteins, however the inclusion of amidosulfobetaines can enhance solubilisation of certain membrane proteins [43].

Despite its limitations 2D-PAGE is still used as a standard tool in the analysis of microbial proteomes. The idea being to first identify the protein complement of the microbe under normal conditions, then subject the organism to a stress stimulus so that the differential expression of proteins can be visualised by either an increase or decrease in spot intensity or by the appearance/disappearance of spots on the gel [44].

### Multi Dimensional Protein identification Technologies

An alternative to the traditional 2-D PAGE technology for microbial proteome analysis is the high throughput approach of multidimensional liquid chromatography coupled to tandem mass spectrometry [34]. In its early stage of development this process was used very successfully for the proteome analysis of the *Saccharomyces cerevisiae *ribosome allowing the identification of more than 100 proteins in a single 24-hour run [39]. The process was further developed and led to the multidimensional protein identification technology (MUDPIT) [45]. A MUDPIT experiment entails the following: A reduced, alkylated and tryptically digested mixture of proteins are separated by first running the peptide mixture on a strong cation exchange (SCX) chromatography column. This solution is then separated into several discrete fractions by a series of wash steps with an increase in salt molarity at each step. The peptides eluted at each salt wash step are then run onto a reverse phase C_18 _column where they are further separated and resolved. The resolved mixtures are then passed directly into the mass spectrometer where tandem mass spectrometry profiles are generated for each peptide; this data is automatically trawled against protein databases for identification. Finally, any novel peptides not in the database can be subjected to *de novo *sequencing [26,45].

This process, whilst seemingly complicated, is highly automated with high throughput achieved in a short time. Washburn and co-workers [45] using this process were able to identify1484 proteins from the *Saccharomyces cerevisiae *proteome in a single twenty-seven hour run. MUDPIT can be seen as complimentary to 2D PAGE as it overcomes many of the problems and limitations of this technique, identifying proteins with extreme pI, integral membrane proteins and low abundance proteins [45].

### Intact cell mass spectrometry

Intact cell mass spectrometry (ICMS) can be employed in microbiology for the rapid analysis, identification and subtyping of specific microorganisms. The use of MALDI-TOF-MS allows the examination of specific peptides or proteins that desorb from intact viruses, bacteria and microbial spores, thus generating peptide mass fingerprints that are unique to the individual microorganisms [46,47]. Walker *et al *[46] assessed ICMS for the identification and subtyping of methicillin-resistant *Staphylococcus aureus *(MRSA) investigating the effects of different culture media and the intra- and inter-laboratory reproducibility of their results in previously characterised isolates of staphylococcal species. Shah *et al *[48] used MALDI-TOF-MS analysis on intact cells of human pathogens to give specific spectral profiles which could be used to delineate bacterial species. Cells were then lysed and subjected to Surface-enhanced laser desorption/ionisation time of flight mass spectrometry (SELDI-TOF-MS): this is a modification of MALDI-TOF-MS in which the stainless steel target plate is replaced by a protein chip array. The chip has a number of sample wells each containing a different chemistry, thus specific classes of molecules may be captured from cell lysates and selectively analyzed. Using this process several toxigenic and nontoxigenic strains of *Bacteroides fragilis *were analyzed revealing potential biomarkers specific to the toxigenic strains in the mass range 3.5–18.5 kDa.

### Expressional Proteomics

Whilst techniques described thus far provide the microbiologist with an invaluable snapshot of the processes occurring within a biological system, assessing the quantitative change in protein expression patterns remains the focus for those interested in the fundamental analysis of microbial systems. There are presently several methodologies that attempt to provide quantifiable expressional analysis. These include the label free emPAI technique; the label based ICAT, iTRAQ and metabolic labelling as well as the gel-based differential in gel electrophoresis (DIGE).

The exponentially modified protein abundance index (emPAI) is a label free methodology for estimating absolute protein abundance in a sample. This methodology is a simple calculation that utilises the output information obtained when tandem mass spectrometry data is processed through database search engines [49].

**Figure 5:**
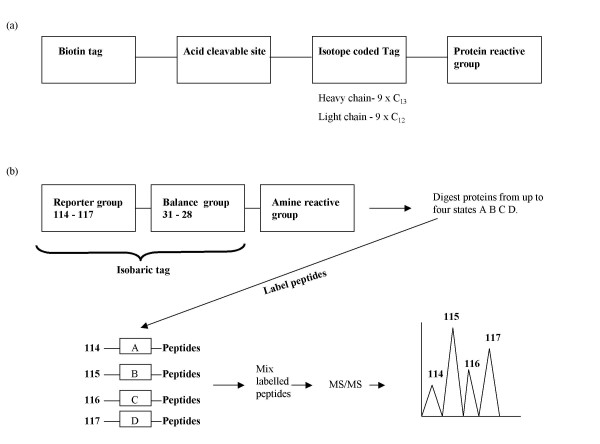
(a) Structure of the isotope coded affinity tag (ICAT). (b) Simplified version of the structure and protocol using the isobaric tag for relative and absolute quantitation (iTRAQ).

The aim of any labelling strategy is to derivatize all proteins/peptides in a sample to allow their analysis [50]. Gygi and co-workers were the first to utilise isotope coded affinity tagging (ICAT) for differential protein expressional analysis of *Saccharomyces cerevisiae *when utilising either galactose or ethanol as a carbon source [51]. The original ICAT reagent consisited of an affinity tag (biotin), to allow labelled peptides to be removed from a mixture by attachment to an avidin column ; an isotopically labelled linker region which was either 'light' containing eight hydrogen atoms d0 or 'heavy' containing eight deuterium atoms d8; and a thiolate-reactive group that allowed labelling of cysteinyl groups [51,52]. Protein mixtures from the two states were labelled separately one with the light reagent and one with the heavy. The two samples were then mixed, tryptically digested and the labelled peptides were separated from the unlabelled by running the sample on an avidin column which binds to the biotin tag. The biotin is then removed and the sample separated on a C_18 _column before analysis on a mass spectrometer. The relative abundance of the light and heavy versions of the peptides can then be compared and information on the protein expressional changes can be identified [51,52]. The present form of the ICAT reagent differs slightly form the original. It contains the biotin affinity tag which is attached to an acid cleavable linker, making it easier to remove; the light and heavy isotopically labelled region contains either nine C_12 _or nine C_13 _atoms (Figure 5a), these overcome slight differential elution problems that were observed when using hydrogen and deuterium; the thiol-specific labelling group remains the same [52,53].

The latest reagent for use in protein labelling, which was utilised by Ross and co-workers to analyse the global protein expression in a wild type *Saccharomyces cerevisiae *and two isogenic mutant strains, is the amine reactive isobaric tag for relative and absolute quantitation (iTRAQ) [54]. The iTRAQ reagent has several advantages over ICAT; four or, in the most recent version, eight states rather than two can be measured; and free amine groups rather than reduced cysteines, which are only present in 95% of proteins, are labelled. The 4-plex reagent contains an amine specific reactive group, a balance group and a reporter group that can have a mass of 114, 115, 116 or 117. Samples of proteins from up to four states are first trypsinised resulting in a peptide mixture with each cleaved peptide having a free amine group. Each sample then is labelled with one of the specific reagents by attachment of the label via the amine specific reactive group. All four samples are then mixed, separated by liquid chromatography and introduced into the mass spectrometer. During tandem mass spectrometry of the labelled peptides the reporter group is released, and measurement of the peak areas of these resultant ions gives an assessment of the abundance of that particular peptide under each condition (Figure 5b) [54].

Metabolic labelling offers one of the most comprehensive methods of investigating microbial proteomes. Unlike other labelling technologies samples to be analysed can be combined before protein extraction thus removing the main source of sample variation, which is the protein extraction process itself [50]. The simplest methodology involves comparison of 'normal' and 'stress' states by growth of the microorganism on media enriched with N^15 ^for one state and on media containing the naturally abundant isotope N^14 ^for the other state. The ratio of N^14^/N^15 ^containing proteins from the two conditions are measured and changes in protein expression levels can be identified [50,52]. This methodology has been successfully utilised by Washburn and co-workers when working on *Saccharomyces cerevisiae *[55]. Additional isotopic variants such as deuterium and C^13 ^enriched media can also be utilised for metabolic labelling [56,57].

Conventional comparative 2D-PAGE requires the production of two gels, one for each condition being compared. Several inherent problems with this approach include the fact that there can be slight variation in the composition of the gels thus giving rise to slight variations in the proteomic profile observed, also sample loading errors can give rise to similar misleading results. Subsequently several replicate gels must be produced in order to obtain as accurate a picture of the proteome as possible [58]. The most efficient way to overcome these problems would be to analyse the different protein samples on one gel. This can now be achieved utilising a technique known as differential in gel electrophresis (DIGE) [59]. In this methodology each protein sample is labelled with one of three structurally similar but spectrally distinct fluorphores that are N-hydroxy-succinymidyl esters of the cyanin dyes Cy2, Cy3 and Cy5. The samples to be investigated are mixed together and run on a 2D-PAGE gel. The resultant gel is then imaged using filters specific to each fluorphor. The ratio of the different signal intensities can be used to determine changes in observed protein expression patterns [58].

### Post -translational modifications

Genomic data alone gives no information on post-translational modification events that occur within many proteins as they are converted to their mature forms. There are currently over 200 reported post-translational modifications [60] the vast majority of these are found in eukaryotic systems. Many of these modifications are regulatory in nature as exemplified by phosphorylation events [14,38]. Phosphorylation of proteins is a key post-transaltional modification that governs the activity of a number of biochemical pathways and enzymatic activities, [38,61] although these phosphorylation cascades are widespread in eukaryotic systems, prokaryotic proteins are also phosphorylated, markedly in the phosphorylation cascade of bacterial two-component signal transduction systems [62]. Therefore detection and identification of this post-translational modification within microbial proteomic investigations is highly desirable as it allows further understanding and elucidation of the processes occurring within a given system. Various methodologies have been developed for investigation of protein phosphorylation.

Phosphoproteins can be identified on 2 D PAGE by the incorporation of radiolabelled orthophosphate into proteins with identification by autoradiography [63]. This method has limitations; it can only be carried out *in vivo *and background staining of DNA and RNA occurs [62]. An alternative to this method is the staining of immunoblots from 2D PAGE with phosphoamino specific antibodies [63]. These antibodies work well for phosphotyrosine but are less effective in the identification of phosphoserine and phosphothreonine [62,64].

An advantage of 2D PAGE over gel-free approaches is that post-translational modifications will cause a mass change and subsequently a shift in the pI of proteins that have been modified, thus the modified and parent proteins usually appear on the gel as horizontal or vertical sets of spots [38,65]. This has been used to identify parallel profiles of phosphoylated and total proteins within a 2D PAGE gel; using sequential staining the gel is first stained with Pro Q™ Diamond phosphoprotein stain and then imaged. The gel is then stained with Sypro Ruby which reacts with all proteins present, the gel is then re-imaged and the two images compared. The phosphorylated proteins, with their increased negative charge, migrate to a more acidic region of the gel compared to the parent protein. This pattern can then be visualised and proteins identified. A multiplexing approach can then be taken by running several gels and comparing the profiles [66]. Another useful method for identification of phosphorylation sites by 2D PAGE is to run samples before and after chemical or enzymatic removal of phosphate groups. In this case spots relating to phosphorylated proteins will disappear from the gel [38,67].

Various gel-free mass spectrometry based protocols have also been developed for the identification of phosphoylation sites on peptides and proteins [61]. However, the analysis of protein phosphorylation is complicated by the fact that these proteins are present in low concentrations and are poorly ionisable[14,61]. In order to better study these post-translationally modified proteins from a proteomic sample one must try to either enrich the intact phosphoproteins or their derived phosphopeptides form the sample to be investigated.

Phosphoprotein enrichment is usually achieved by immunoprecipitation using antibodies directed against phosphotyrosine [68]. However, a more commonly used approach, which gives a more global overview in studying the phosphoproteome, is the enrichment of phosphopeptides. Enrichment can be achieved with IMAC as demonstrated by Ficarro et al [69] studying the phosphoproteome of *Saccharomyces cerevisiae *and can also be achieved utilising graphite [70] and titanium oxide [71]. This approach both reduces the complexity of the sample to be analysed and allows you to gain specific information on the sites of phosphorylation.

Once the phosphoproteome has been enriched mass spectrometry techniques can be used to identify the sites of phosphorylation. These include neutral loss scanning for the loss of the 98 Da phosphoric acid moiety, which is lost from phosphoserine and phosphothreonine during CID in ion trap mass spectrometers. This method cannot be used to detect phosphotyrosine as there is no loss of a phosphoric acid moiety during CID [68]. However, another technique, parent ion scanning in positive ion mode searching for the immonium ion of phosphotyrosine at m/z 216.043 can be used for its identification. This technique utilised in negative ion mode for an ion of m/z 79 (corresponding to PO_3_^-^) can also be used for the identification of phosphoserine and phosphothreonine [72].

### Some important considerations for proteomic data analysis

Even if researchers invest considerable time and effort in establishing a robust proteomic workflow for their microorganism of choice, this good work can be undone if due care and consideration is not given to a number of key aspects in data acquisition, processing and analysis:

#### Replicate Injections

Due to the complexity of peptide mixtures within a proteomic sample the separation capabilities of LC-MS systems are often exceeded. This, coupled to the limitations of the data dependent acquisition for the selection of peptides for MS/MS, requires that samples be run more than once in order to gain as wide a proteome coverage as possible [73-75]. During a proteomic investigation of *E*. *coli *by Taoka and colleagues [76] ten repeated injections of the same sample was carried out and showed that the number of new proteins identified in each run increased until the third and fourth run, where further injections did not greatly increase the number of proteins identified. This suggests that researchers must perform at least triplicate analysis of the same fraction and recent studies have shown that such an approach leads to an increase in the number of proteins identified by up to 40% [73-75].

#### Data curation

Large scale proteomic investigations generate huge amounts of raw data, in some experiments up to 2 GB per run [26]. This presents a considerable problem for the conscientious researcher. In order to glean any meaningful biological information from this raw data the researcher must in some way curate it. The MS/MS spectra from individual peptides from an LC-MS/MS run can be analysed utilizing a number of bioinformatic tools, with the probability-based tool, MASCOT, being the most widely used [77]. The experimental mass values generated by MS/MS of peptides from the sample under investigation are compared with precalculated peptide mass or fragment ion mass values, that have been obtained by applying specific cleavage rules to the primary sequence entries of proteins contained within a database of interest. By using an appropriate scoring algorithm, the closest match or matches can then be identified. Previously we have reported on the limitations of current automated MS data interpretation from large-scale proteomics studies [73-75]. One of the major challenges that we encountered was the elimination of erroneous data that may lead to the identification of 'phantom' proteins within any given sample. To this end we suggested that until a reliable MS data interpretation tool could be found, the only way to proceed, and ensure integrity of data interpretation, was to manually curate the MS data, resulting in many laborious weeks of analysis. For example when working with the subproteome of *Geobacillus thermoleovorans *we reported a total processing time of 40 days, whilst Chong & Wright [73] working with similar datasets from *Sulfolobus solfataricus *P2 took over 43 days. This represents a bottleneck in the proteomic workflow and a not inconsiderable effort by researchers involved. In order to tackle this problem reliable bioinformatics tools for curation of data may be used. There are many such tools but in the course of our research, to expedite the curation of the identified protein list from MASCOT [77], we utilize the protein validation tool PROVALT [78]. The result files from the MASCOT search are reanalyzed with PROVALT. This automated program takes large proteomic MS datasets and reorganizes them taking the multiple MASCOT results and identifies peptides that match. Redundant peptides are removed, and related peptides are grouped together associated with their predicted matching protein; the program dramatically reduces this portion of the curation process [75,79].

Another consideration when publishing proteomics data is the use of randomized databases in order to attempt to make a statistical analysis of the number of false positive identifications. These false positives can be generated due to the peptides identified in the MS analysis randomly matching entries in the databases giving positive identifications, when in fact the peptide did not actually originate from that protein. Almost all of the major proteomic journals, led by Molecular and Cellular Proteomics, require some information on false positive rates for large proteomic datasets. Once again there are many methodologies for assessing this rate, in our research, however, PROVALT can carry out this analysis automatically.

PROVALT uses peptide matches from a random database to calculate false-discovery rates (FDR) for protein identifications. Identifications from searching the normal and random databases are used to calculate the FDRs and set score thresholds and thus identify as many 'actual' proteins as possible while encountering a minimal number of false-positive protein identifications. Rather than calculate error rates at the peptide level, the FDR calculations employed by PROVALT provide a reasonable balance between the number of correct and incorrect protein assignments. The FDR is usually set at 1%, meaning that 99% of the reported proteins identified should be correct [75,78,79].

Additional functional information can be achieved for the generated protein list from any proteomic investigation by attempting to assign these proteins to specific cellular localization. This is important in allowing researchers to identify those proteins that are retained and exported from cells. It also has the potential commercial application of enabling the identification of possible diagnostic and therapeutic targets. Currently a number of bioinformatics tools are available for this task including PSortB [80], SignalP [81] and SecretomeP [82]. These attempt to assign a subcellular location for each protein, based upon the prediction of known motifs or cleavage sites, through the use of a variety of computational algorithms and networks that analyse their amino acid composition. Researchers are now however moving toward "smarter" *in silico *strategies whereby a number of predictors based on both structural and experimental data are being used to attempt to predict protein localization [83]. Until this next generation of bioinformatics tools are widely available, the researcher must manually interpret the results to gain any level of biological significance.

### Microbial proteomic case study: The Bacillaceae

Reliable and detailed genomic information is required in order for scientists to realize the maximum potential of proteomics to assist in our understanding of how specific microorganisms function under a given condition. The Bacillaceae are one group of bacteria particularly well represented within the Comprehensive Microbial Resource database at The Institute for Genomic Research [84] who give details on the completed genome sequencing projects for 20 members of this family, including: *Bacillus anthracis *(9 strains); *B*. *cereus *(3 strains); *B*. *licheniformis *(2 strains); *B*. *subtilis*; and *B*. *thuringiensis *konkukian. The availability of such rich genomic information allows a systems biology approach to be taken when investigating the Bacillaceae. Work on *Bacillus subtilis *by Michael Hecker and his group at the University of Greifswald can be viewed as an exemplar in this field. In a series of well crafted studies Hecker has successfully exploited genomics and advances in proteomics to provide insights into the physiology and metabolism of *B*. *subtilis *under a myriad of conditions ranging from salt stress to nutritional starvation [85]. Most recently this group has adopted a combined gel based and gel free approach to further 'drill down' into the proteome of this important model organism [86-89].

Whilst the aforementioned bacterial species represent a number of organisms of major importance in medical and food research, it is perhaps within the genomes of extremophilic bacilli that discoveries of significance for biotechnology and evolutionary theory will be made. Genome sequences have been completed by both commercial and government agencies within Japan for thermophilic, alkaliphilic and halophilic organisms represented by *Oceanobacillus *iheyensis HTE831 [90], *B*. *clausii *KSMK16, *B*. *halodurans *C-125 [91] and *Geobacillus kaustophilus *HTA426 [92]. The commercial relevance of such organisms can be seen in the industrial interest that has arisen in *Geobacillus *species with their potential applications in biotechnological processes, for example as sources of various thermostable enzymes, such as proteases [93], amylases [94], lipases [95], and pullanases [96].

Our own group has a strong interest in such organisms and has reported the first global proteomic analysis of the soluble and insoluble subproteomes of the highly thermophilic aerobic eubacterium, *Geobacillus thermoleovorans *T80 [74]. This work allowed us to gain insight into cellular processes within the cytosol of this bacterium, for example, we identified a number of sigma factors, such as *ó*A, that initiate transcription of the heat shock operons controlled by the HrcA-CIRCE complex within Gram-positive bacteria. In addition within the insoluble subproteome we could identify membrane-associated proteins, secreted proteins, and those integral to the membrane, with functionalities including transport, osmoregulation, and heat shock response [75].

With the prevalence of genomic data for *Bacillus *and related genera, it has become possible to use advanced bioinformatic approachs to propose hypotheses on how these organisms adapt to extreme environmental conditions. Takami et al. employed a comparative genomic analysis of the alkaliphilic and extremely halotolerant *O*. *iheyensis*, the alkaliphilic and moderately halotolerant *B*. *halodurans *and the neutrophilic and moderately halotolerant *B*. *subtilis *to identify a number of candidate genes of importance in adaptation to highly alkaline environments [97]. Recently we described the first proteomic investigation of *O*. *iheyensis *[98] which allowed us to identify, at neutral pH, a number of proteins belonging to two putative transport systems believed by Takami et al [97] to be of importance in the alkalaphilic adaptation of *Oceanobacillus iheyensis *HTE831. Our observations reaffirm the necessity of postgenomic expression studies to validate hypotheses generated via comparative genomic analysis of this and presumably other organisms.

### MetaProteomics

Microbial derived commodities or processes can often be as a result of mixed populations of organisms, the composition of which can vary and is often far from being highly defined, rather than by single axenic cultures. Whilst ongoing studies aim to catalog such systems by use of a plethora of techniques aimed at the indentification of species diversity this is of relatively limited use when it is remembered that functional output is more important than compositional diversity. This point is extremely well illustrated by the work of Fernandez *et al*. [99], who analysed the community dynamics of a functionally stable and well-mixed glucose fed methanogenic reactor over a period of 605 days. Analysis of the distribution of operational taxonomic units (OTUs) generated from a range of time-point samples revealed a chaotic pattern. It was proposed that this extremely dynamic community structure observed within the bioreactors helped maintain a stable ecosystem function. Under such circumstances analysis of the catalytic potential of the bioreactor, by detection of the total protein content present, would give a more simplistic and relevant 'picture' of system functionality.

In order to investigate such 'black box' systems transcriptomics has been shown to be a useful tool that can be applied to help understand the complex interactions occurring within microbial communities [99]. Limitations of this approach, however, have recently been reviewed and include information bias due to the necessary selection of specific genes for microarray construction and the increasing evidence for the lack of direct correlation between mRNA expression and protein expression [100]. Metaproteomics, an emerging field that aims to analyse the proteins of mixed microbial communities, offers a complimentary and enhancing tool for transcriptomics and is therefore of great potential to those interested in understanding the functionality of mixed microbial systems.

The benefits of this approach are best illustrated by the work of three groups in the metaproteomic vanguard. Paul Wilmes and Phil Bond working at the University of East Anglia developed 2D-PAGE methods for the proteomic analysis of a mixed microbial community involved in enhanced biological phosphorus removal [101]. This work was further developed to allow analysis of two laboratory-scale sequencing batch reactors with dissimilar phosphorus removal performances [102]. Metaproteomic analysis of these systems found that protein expression pattern was fairly stable within the reactor with best phosphorus removal capability compared with its poorly functioning companion reactor. Whilst the study is still in its infancy it has allowed Wilmes and Bond to suggest that a bioenergetic advantage is available to the optimally functioning reactor due to its equilibrated protein expression [103].

Schulze and coworkers at the University of Southern Denmark have also applied proteomic fingerprinting to the analysis of dissolved organic matter from four different environments and thus identify the potential catalytic function of proteins within these ecosystems [104]. This work revealed that enzymes involved in the degradation of organic matter could not be found in free soil dissolved organic matter. Rather enzymes such as laccases and cellulases could be detected in proteins extracted from soil particles that may indicate that degradation of soil organic matter only takes place within biofilms located on particle surfaces [104].

Finally the most extensive metaproteomic analysis to date was carried out on a natural microbial biofilm by Jillian Banfield's group based in the University of California at Berkeley. Ram et al [105] described the detection of 2033 proteins within the proteome of a biofilm growing inside a mine in which the physiochemical conditions of the biotope included low pH (~0.8), a relatively mesophilic temperature (~42°C) and the presence of high concentrations of heavy metals. The stringent curation of proteomic data utilised by this group led to the identification of 48% of the predicted proteins from the most abundant biofilm member, a Leptospirillium group II. Those highly expressed proteins detected within the biofilm included those involved in oxidative stress response giving an insight into how the microbial biofilm survives. A major benefit of metaproteomics is the removal of the need to preselect the gene products thought to be present in the environment being investigated as is necessary for transcriptomic investigations. This removal of bias allowed Ram et al [105] to detect an abundant and novel cytochrome that was central to iron oxidation and actual formation of the biofilm.

## Conclusions and future directions

The generation of large genomic datasets is revolutionising both our understanding of microbiology and the way in which we as microbiologists investigate the organisms that interest us. Systems biology has been revitalized by this expansion in genomic information and its exponents now seek to explain complex biological systems in terms of their molecular components and their interactions. Microorganisms are therefore ideal candidates for systems biology research and the field of systems microbiology is expected to result in the development of tools and techniques of general applicability across the life sciences [106].

Proteomics has a key role to play in attempts to construct comprehensive cellular maps of biochemical processes occurring within specific microorganisms at given spatial and temporal points. Proteomic data when coupled with bioinformatic programs such as the BioCyc database [107], a collection of 160 pathway/genome databases for most eukaryotic and prokaryotic species whose genomes have been completely sequenced, allows a virtual model of biochemical pathways within a microorganism to be constructed. It is then possible to begin to model and possibly regulate microbial cellular metabolic pathways and processes in order to maximise production of commodities or to identify new drug targets. Indeed the experimental synthesis of pathways predicted by systems biology to exhibit novel properties has resulted in the development of a new and exciting field of study, termed as synthetic biology [106,108]. An understanding of the complexities and possibilities offered by mass spectrometry is essential for those researchers who wish to further both our understanding of, and methods for studying microbial proteomics and to fully exploit the exciting applications of systems and synthetic biology.
